# Osteopontin predicts late-time salience network-related functional connectivity in multiple sclerosis

**DOI:** 10.1371/journal.pone.0309563

**Published:** 2024-08-29

**Authors:** Zsofia Kakucs, Zsolt Illes, Zsofia Hayden, Timea Berki, Gergely Orsi

**Affiliations:** 1 Department of Medical Imaging, Medical School, University of Pecs, Pecs, Hungary; 2 Department of Radiology and Medical Imaging, Mures County Emergency Clinical Hospital of Targu Mures, Targu Mures, Romania; 3 Department of Neurology, Medical School, University of Pecs, Pecs, Hungary; 4 Department of Neurology, Odense University Hospital, Odense, Denmark; 5 Department of Clinical Research, University of Southern Denmark, Odense, Denmark; 6 Department of Immunology and Biotechnology, Medical School, University of Pecs, Pecs, Hungary; 7 HUN-REN-PTE Clinical Neuroscience MR Research Group, Hungarian Research Network, Pecs, Hungary; University of Rijeka Faculty of Medicine: Sveuciliste u Rijeci Medicinski fakultet, CROATIA

## Abstract

Resting-state functional magnetic resonance imaging (rs-fMRI) has been widely utilized to investigate plasticity mechanisms and functional reorganization in multiple sclerosis (MS). Among many resting state (RS) networks, a significant role is played by the salience network (SN, ventral attention network). Previous reports have demonstrated the involvement of osteopontin (OPN) in the pathogenesis of MS, which acts as a proinflammatory cytokine ultimately leading to neurodegeneration. Concentration of serum OPN was related to MRI findings 10.22±2.84 years later in 44 patients with MS. Local and interhemispheric correlations (LCOR, IHC), ROI-to-ROI and seed-based connectivity analyses were performed using serum OPN levels as independent variable along with age and gender as nuisance variables. We found significant associations between OPN levels and local correlation in right and left clusters encompassing the central opercular- and insular cortices (p-FDR = 0.0018 and p-FDR = 0.0205, respectively). Moreover, a significant association was identified between OPN concentration and interhemispheric correlation between central opercular- and insular cortices (p-FDR = 0.00015). Significant positive associations were found between OPN concentration and functional connectivity (FC) within the SN (FC strength between the anterior insula ventral division and 3 other insular regions, F(2,13) = 7.84, p-FDR = 0.0117). Seed-based connectivity analysis using the seven nodes of the SN resulted in several positive and inverse associations with OPN level. Serum OPN level may predict FC alterations within the SN in 10 years.

## Introduction

Multiple sclerosis (MS) is a chronic neuroinflammatory and neurodegenerative disease of the central nervous system (CNS), the leading cause of nontraumatic neurological disability in young adults [1].

Resting-state functional magnetic resonance imaging (rs-fMRI) has been widely utilized in recent years to advance our understanding of the pathophysiology of MS, allowing us to investigate plasticity mechanisms and functional reorganization in the MS population. Identification of these structural and functional network changes continues to be crucial in order to decipher the source of complex conditions like cognitive impairment, depression and physical disability [2].

While the default-mode network (DMN) is a key focus in MS research, clinically relevant functional connectivity (FC) changes extend beyond the DMN [3–5]. Previous studies have highlighted the involvement of many resting-state (RS) networks, with a significant role played by the salience network (SN, ventral attention network) [6]. The main components of the SN are the anterior insula and the dorsal anterior cingulate cortex, which are involved in directing the information flow and modulating the balance between DMN and the central executive network (CEN) [7]. The SN is responsible for the recognition of salient environmental stimuli, ensuring normal network efficiency during different tasks [8].

Osteopontin (OPN), also known as early T cell-activation gene 1 or secreted phosphoprotein 1 (SPP1), was originally identified as an immobilized extracellular matrix molecule in mineralized tissue [9]. Recent studies have demonstrated the role of OPN in the pathogenesis of MS, where it functions as a proinflammatory cytokine, contributing to the amplification of the immunological cascade and ultimately leading to neurodegeneration [10].

In our previous study, we found a strong correlation between OPN concentration measured from the cerebrospinal fluid (CSF) and late time atrophy markers (increased CSF and ventricular volumes along with reduced regional brain volumes in both gray- and white matter) in MS patients [11].

Moreover, we observed a significant association between CSF OPN levels and microstructural alterations in the normal appearing white matter (NAWM) and total white matter lesion volume in the brain a decade later (10.22±2.84 years). Furthermore, serum OPN levels showed numerous associations with FC changes between the nodes of DMN [4].

In this current study, building upon previous findings in MS [6], we aimed to assess the association between OPN levels and FC alterations within the SN.

## Materials and methods

### Subjects and samples

Fourty-four patients (32 females, mean age 42.8 years [range 20–68]) were prospectively recruited for the study. Inclusion criteria were definitive MS diagnosis according to the 2017 modified McDonald diagnostic criteria [12], available serum sample stored in the Institutional Serum and Liquor Bank collected at least 5 years earlier. The median EDSS of the included patients was 2, the disease course distribution was as follows: relapsing-remitting MS (RRMS) 68% of which 12 patients were classified as benign RRMS, primary progressive MS (PPMS) 11%, secondary progressive MS (SPMS) 21% (Table 1). RRMS-SPMS conversion occurred between the initial serum collection and the MRI measurement in seven patients, while in case of an additional patient, the conversion was observed around the initial sample collection.

**Table 1:** Clinical characteristics of MS patients.

Characteristics	Number of patients, mean±SD or median(IQR)
Demographics	
Number of patients	44
Age (years)	42.8±7.9
Disease duration (years)	11.2±3.0
Age at onset (years)	31.7±6.7
Sex (female/male)	32/12
Years between serum collection and MRI	10.2±2.8
Disease type at the time of MRI (number of patients)	
PPMS	5 (11%)
SPMS	9 (21%)
RRMS	30 (68%)
of which benign RRMS	12
EDSS	
At time of initial serum collection	2(1–2)
At time of MRI	2(0.25–5.25)
DMT at the time of MRI	
None	14 (32%)
Interferon-beta	11 (25%)
Glatiramer acetate	6 (14%)
Fingolimod	4 (9%)
Dimethyl fumarate	4 (9%)
Teriflunomide	3 (7%)
Ocrelizumab	1 (2%)
Alemtuzumab	1 (2%)
OPN serum concentration at time of initial serum collection	
Mean concentration (pg/ml)	13954.2±6843.0
PPMS	11328.5±2531.7
SPMS	24456.3±13917.6
RRMS	15678.3±2307.9
RRMS benign	12393.5±7558.9
OPN serum concentration at time of MRI	
Mean concentration (pg/ml)	14597.9±10611.1
PPMS	8890.5±6629.4
SPMS	13555±8850.6
RRMS	17930.6±13868.4
RRMS benign	13036.9±6455.9

Normally distributed data are reported as mean±SD, non-normally distributed data are reported as median (25–75% interquartile range). SD: standard deviation, IQR: interquartile range, PPMS: primary-progressive multiple sclerosis, SPMS: secondary-progressive multiple sclerosis, RRMS: relapsing-remitting multiple sclerosis, EDSS: Expanded Disability Status Scale, CSF: cerebrospinal fluid, DMT: disease modifying therapy, OPN: osteopontin.

The study was conducted according to the World Medical Association Declaration of Helsinki and approved by the Regional Ethical Committee of the University of Pecs (7068-PTE 2018). All patients signed written informed consent.

Serum samples were collected 10.22±2.84 years before MRI, and aliquots were kept at −80°C until processing. A commercially available enzyme-linked immunosorbent assayhttps://www.sigmaaldrich.com/US/en/products/protein-biology/immunoassay-platform-solutions/elisa-kits (ELISA) kit (Human Osteopontin DuoSet ELISA, R&D Systems, Minneapolis, MN) was used, according to the manufacturer’s instructions. All samples were run in duplicates. Optical density was detected at 450 nm using an iEMS MF microphotometer (Thermo Labsystem, Beverly MA, USA).

### Magnetic resonance imaging

Subjects were scanned using a 3T MRI scanner (MAGNETOM Prisma^Fit^, Siemens-Healthineers, Erlangen, Germany). The MRI study protocol included the following sequences: 3D T1 magnetization-prepared rapid acquisition with gradient echo (MPRAGE), 3D fluid-attenuated inversion recovery (FLAIR), and rs-fMRI with field mapping to reduce image distortions due to B0 inhomogeneities. Imaging parameters are disclosed in the *Supplementary materials*.

### rs-fMRI evaluation

Evaluation was carried out by using CONN (21.a) [13] and SPM (12.7771) [14]. All preprocessing and denoising steps were done using the recommended default settings, except for the following steps: voxel displacement maps were created for distortion correction, EPI-normalization was switched to indirect method, and final Gaussian smoothing was reduced to 6 mm full-width at half-maximum (FWHM).

First-level analyses included local correlation map (LCOR) calculations estimated as the weighted average of all short-range connections between a voxel and a 25 mm FWHM Gaussian neighbourhood area, interhemispheric correlation map (IHC) calculations characterizing the strength of homotopic FC between the two hemispheres, ROI-to-ROI connectivity analysis characterizing the FC between each pair of regions, and seed-based connectivity maps estimation characterizing the spatial pattern of FC with a seed area.

ROIs of the SN were defined by the MIST122 atlas [15]. Details of pre-processing, denoising, and group analyses are elaborated in the *Supplementary materials*.

### Statistical analysis

For each individual voxel (or connection in case of ROI-to-ROI analysis) a separate GLM was estimated, with first-level connectivity measures at this voxel (or connection) as dependent variable and osteopontin level, age, and gender as independent variables. Storage time was also included as covariate of no interest, but was eventually removed from all models, due to the lack of significant contribution. Voxel-level or connection level (in case of ROI-to-ROI) hypotheses were evaluated using multivariate parametric statistics with random-effects across subjects and sample covariance estimation across multiple measurements. Inferences were performed at the level of individual clusters (groups of contiguous voxels or in case of ROI-to- analysis: groups of similar connections). Cluster-level inferences were based on parametric statistics from Gaussian Random Field theory. Results were thresholded using a combination of a cluster-forming p < 0.001 voxel-level threshold, and a familywise corrected p-FDR < 0.05 cluster-size threshold, or in case of ROI-to-ROI analysis: a combination of a p < 0.05 connection-level threshold and a familywise corrected p-FDR < 0.05 cluster-level threshold. Difference in median OPN concentrations between the two timepoints was assessed by Wilcoxon Signed Rank test, correlation between storage time and measured OPN concentration was assessed by Pearson product-moment correlation.

## Results

Clinical characteristics and measured OPN concentrations are presented in Table 1. Median OPN did not differed significantly between the two timepoints (Z = -0.966, p = 0.352).

**Fig 1:**
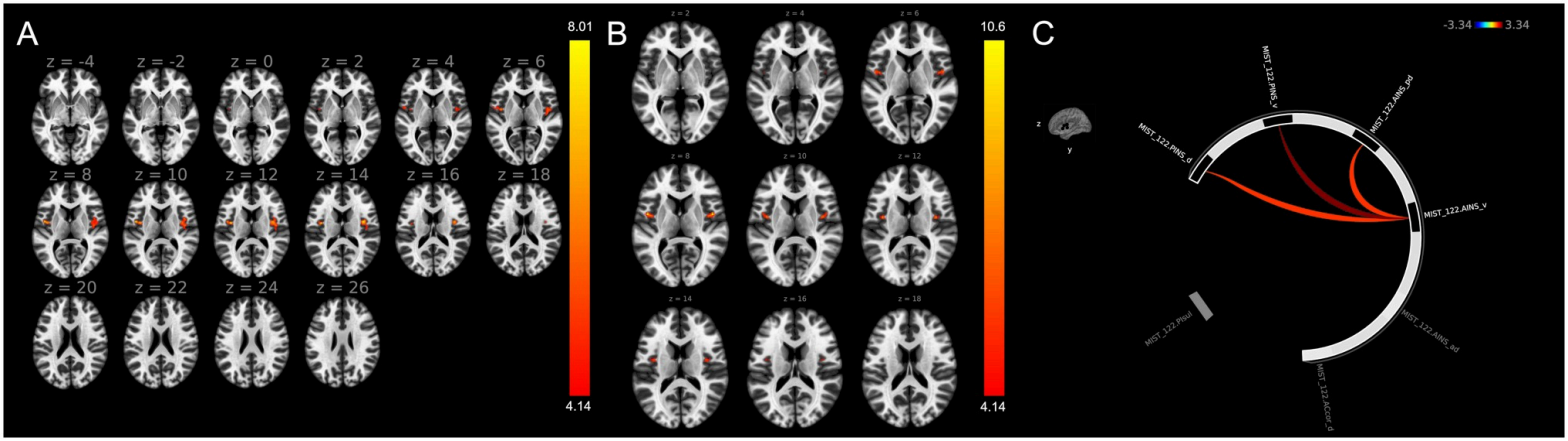
A: Local correlation analysis using serum osteopontin levels as independent variable along with age and gender as nuisance variables. Significant clusters are shown bilaterally in the central opercular and insular cortices. B: Interhemispheric correlation analysis using serum osteopontin levels as independent variable along with age and gender as nuisance variables. Significant associations are shown bilaterally in the central opercular and insular cortices. C: ROI-to-ROI analysis in the salience network. Significant associations were observed between OPN concentration and FC strength between the AINS_v and 3 other insular regions (AINS_pd, PINS_d, and PINS_v). Abbreviations: ACcor_d: Anterior Cingulate Cortex dorsal division; AINS_ad: Anterior Insula anterodorsal division; AINS_pd: Anterior Insula posterodorsal division; AINS_v: Anterior Insula ventral division; PINS_d: Posterior Insula dorsal division; PINS_v: Posterior Insula ventral division; PI_sul: Periinsular sulcus. Part A and B: Random field theory parametric statistics (voxel threshold p < 0.001, cluster threshold p < 0.05, cluster-size p-FDR corrected). Images are shown in radiologic convention. Part C: Parametric multivariate statistics. Cluster threshold p < 0.05, cluster-level p-FDR corrected (MVPA omnibus test) connection threshold p < 0.05.

LCOR analysis using serum OPN levels as independent variable along with age and gender as nuisance variables resulted in significant association between OPN levels and local correlation in right and left clusters encompassing the central opercular- and insular cortices (p-FDR = 0.0018 and p-FDR = 0.0205, respectively), see Fig 1A.

IHC analysis using serum osteopontin levels as independent variable along with age and gender as nuisance variables resulted in significant association between OPN levels and interhemispheric correlation between central opercular- and insular cortices (p-FDR = 0.00015), see Fig 1B.

Analysis was conducted between the nodes of the salience network defined using the MIST122 atlas. The following seven labels were used for the analysis: anterior cingulum dorsal part (ACcor_d); posterior insular cortex ventral and dorsal divisions (PINS_v, PINS_d); anterior insular cortex anterodorsal, posterodorsal, and ventral divisions (AINS_ad, AINS_pd, AINS_v); and periinsular sulcus (PIsul). Significant positive associations were found between OPN concentration and FC strength between the AINS_v and 3 other insular regions (AINS_pd, PINS_d, and PINS_v, F(2,13) = 7.84, p-FDR = 0.0117), see Fig 1C.

Seed-based connectivity analysis using the seven nodes of the SN, defined in the previous section, resulted in several positive and inverse associations with OPN measured from samples taken 10.22 years before the MRI. Results are summarized based on the seed region. Significant regions for ACcor_d seed were right temporal pole and lingual gyrus, while insular seed regions mainly resulted in significant regions in other parts of the insular- and opercular cortices, temporal pole, and postcentral gyrus. Detailed results are listed in Table 2.

**Table 2:** Seed-based connectivity results of the salience network regarding the associations between the osteopontin concentration and FC measured 10.22 years later.

Seed	Cluster index	Structure name	Direction of association	Cluster size	p-FDR	MNI (x,y,z)
ACcor_d	1	RIGHT TEMPORAL POLE	Positive	130	0.000348	48, 20, -26
	2	RIGHT LINGUAL GYRUS	Inverse	78	0.006547	30, -56, 4
AINS_pd	1	LEFT TEMPORAL POLE, PLANUM POLARE	Positive	124	0.000537	-46, 8, -18
	2	LEFT SUPERIOR FRONTAL GYRUS	Inverse	111	0.000634	-12,4,64
	3	RIGHT POSTCENTRAL GYRUS	Positive	103	0.000730	44, -14, 26
	4	LEFT CENTRAL OPERCULAR CORTEX, INSULAR CORTEX	Positive	71	0.005715	-40, -6, 14
	5	RIGHT PLANUM POLARE, TEMPORAL POLE	Positive	67	0.006271	48, 2, -12
	6	LEFT FRONTAL POLE	Positive	64	0.006651	-16, 60, 18
	7	PRECUNEOUS	Inverse	60	0.007909	-2, -50, 50
	8	RIGHT CENTRAL OPERCULAR CORTEX, INSULAR CORTEX	Positive	38	0.048891	42, -2, 14
AINS_v	1	RIGHT INFERIOR TEMPORAL GYRUS, TEMPORAL OCCIPITAL FUSIFORM CORTEX, LATERAL OCCIPITAL CORTEX	Positive	252	<0.000001	52, -58, -18
	2	RIGHT POSTCENTRAL GYRUS	Positive	181	0.000007	44, -20, 26
	3	RIGHT LATERAL OCCIPITAL CORTEX	Positive	88	0.001865	44, -74, -2
	4	LEFT TEMPORAL POLE	Positive	86	0.001865	-46, 8, -16
	5	RIGHT PLANUM POLARE, TEMPORAL POLE	Positive	76	0.003238	48, 6, -12
	6	LEFT FRONTAL POLE	Positive	46	0.035124	-18, 58, 18
PINS_d	1	RIGHT CENTRAL OPERCULAR CORTEX, INSULAR CORTEX	Positive	115	0.000819	40, -4, 12
	2	RIGHT POSTCENTRAL GYRUS, CENTRAL OPERCULAR CORTEX	Positive	73	0.008407	44, -14, 24
	3	LEFT INSULAR CORTEX, CENTRAL OPERCULAR CORTEX	Positive	53	0.021700	-34, -4, 10
	4	RIGHT SUPERIOR PARIETAL LOBULE	Inverse	48	0.026982	20, -48, 50
	5	LEFT TEMPORAL POLE, PLANUM POLARE	Positive	44	0.032355	-46, 6, -18
PINS_v	1	RIGHT POSTCENTRAL GYRUS, PARIETAL AND CENTRAL OPERCULAR CORTEX	Positive	173	0.000034	44, -16, 24
	2	PRECUNEOUS	Inverse	93	0.002543	10, -60, 60
	3	LEFT TEMPORAL POLE, PLANUM POLARE	Positive	84	0.003218	-48, 10, -18
	4	RIGHT CENTRAL OPERCULAR CORTEX, INSULAR CORTEX	Positive	77	0.004038	38, -4, 10
	5	LEFT CENTRAL OPERCULAR CORTEX, INSULAR CORTEX	Positive	63	0.009499	-46, -2, 10

Only FDR corrected significant results are listed. MNI coordinates are listed in mm. Abbreviations: ACcor_d: Anterior Cingulate Cortex dorsal division; AINS_ad: Anterior Insula anterodorsal division; AINS_pd: Anterior Insula posterodorsal division; AINS_v: Anterior Insula ventral division; PINS_d: Posterior Insula dorsal division; PINS_v: Posterior Insula ventral division; PI_sul: Periinsular sulcus.

## Discussion

The results of the current study indicate that the functional alterations within the SN are associated with the serum OPN concentration measured approximately 10 years earlier.

OPN is involved in a variety of physiologic functions and pathological conditions such as bone remodeling, wound healing, cancer development, vascular disorders [16]. Previous studies have also pointed out the important role of OPN in the pathogenesis of various autoimmune diseases, such as Sjögren disease [17], inflammatory bowel disease [18], neuromyelitis optica spectrum disease [19] and MS [20]. This pleiotropic cytokine widely expressed by various immune cells, including T cells, B cells, macrophages, dendritic cells, and natural killer cells. OPN promotes the production of pro-inflammatory cytokine by Th1 cells (IL-12, IL-17, IFNγ) and inhibits IL-10 expression by macrophages [21]. OPN also regulates Th17 cell responses and inhibits the TH2 responses [22]. All these pathways are suggested to be involved in the pathogenesis of MS [23].

OPN is expressed in active lesions, especially in acute active MS plaques. Moreover, enhanced OPN expression was found in macrovascular endothelial cells and macrophages in these plaques, as well as in the white matter surrounding the plaques [24]. OPN also interacts with microglial cells, modulating inflammatory responses and attenuating secondary neurodegeneration [25].

According to a meta-analysis including 22 studies, higher levels of OPN were shown to present in peripheral blood and CSF of MS patients compared to the healthy controls (p<0.05) [16]. Moreover, in all types of MS patients, except for those with clinically isolated syndrome (CIS), increased blood levels of OPN were found compared to controls. The concentration of OPN in the CSF was significantly elevated in patients with RRMS compared to those with CIS and SPMS (p<0.05). Additionally, individuals with active MS displayed significantly higher OPN levels in their CSF compared to those with stable disease (p = 0.007).

Similarly, in a recent study, serum OPN levels were significantly higher in RRMS patients (29.48±27.71 ng/ml among patients in remission phase and 23.72±27.6 ng/ml in patients during relapse before receiving treatment) compared to healthy controls (4.1±1.79) [26].

The SN, specifically the right fronto-insular cortex, plays a critical role in switching between internally directed cognition of the DMN and the externally directed cognition of the CEN [27]. The normal functioning of both the DMN and CEN is known to be crucial for fulfilling various cognitive tasks [28]. The compromised connectivity between SN and these two regions can have detrimental effects on cognitive processing. Prior studies have documented varying global connectivity levels of the DMN and the CEN in relation to cognitive impairment in MS. This discrepancy raises questions about how these FC changes can influence cognitive performance. Beyond the directionality of these changes, it remains unclear whether these cognitive disturbances are primarily attributed to modifications in within-network connectivity, connectivity with the broader brain network, or alterations in between-network connectivity.

Compared to healthy controls, MS patients with relapsing-remitting multiple sclerosis (RRSM) presented a decreased RS FC in regions of SN, CEN and DMN. Moreover, increased FC was found between SN and CEN [2]. Recent observations showed disease-stage specific course, even though longitudinal studies evaluating connectivity changes are still rare. A recent study indicates that the centrality of SN increases in the early stages in association with increased FC between the SN and DMN, and between SN and CEN respectively. Conversion from intact cognition to cognitive impairment was related to an initial abnormal function of SN, and this network disturbance was subsequently transferred to DMN. These findings suggest that SN changes may precede DMN dysfunction [6].

The insula comprises anterior and posterior sections: the anterior insula is specifically sensitive to salient environmental events [8]. The involvement of the insular cortex in the pathogenesis of MS has already been demonstrated by several studies. Significant decreases in cortical gray matter (GM) volume were found bilaterally in the insular region of RRMS patients compared to normal controls [29]. The GM volume of the insular lobes are in association with the cognitive scores in MS patients [30]. Moreover, the degree of a key long-range FC of the anterior insula to was related to the measured cognitive reserve index in MS patients [31], while local activation differences within the left insula were also demonstrated in MS patients compared to healthy controls [8]. All the above studies emphasize the importance of insular function in MS pathogenesis. In line with these findings, the results of the current study show that RS insular function within the insula and between bilateral insular regions showed highly significant associations with OPN, which was shown to be involved in MS pathogenesis, typically in the observed long-term structural and functional disturbances [11].

Moreover, the insula and cingulate cortex are predilection locations for cortical demyelinated lesions in MS patients [32]. Thus, any structural and functional change in these sites may result in aberrant SN connectivity.

In summary, the results of the current ROI-to-ROI analysis within the SN shows that part of the functional alterations observed within the SN may be predicted by OPN, suggesting that the functional disturbance of the SN is closely related to MS pathogenesis. Given the main function of the SN, we may hypothesize that the disturbed function of SN could lead to an imbalance between DMN and CEN, associated with altered diversity of structural and functional connections for information flow. This is hypothesis is strengthened by the observations of Huiskapm et al., who concluded that the functional changes within the SN may precede DMN dysfunction [6].

### Limitations

This study has inherent limitations related to its design. The main limitation is the lack of initial MRI measurements. Moreover, this study was conducted at a single center and involved a relatively limited cohort of participants. Previous reports suggest that patients with RRMS during relapse presented higher serum OPN levels than patients with RRMS during clinical remission [10]. In this study, serum samples were obtained in all MS patients regardless of the clinical presentation or MS subtype. Even though there was no correlation between storage time and measured OPN concentrations a decade later (Pearson’s r = 0.026, p = 0.913), neither did the inclusion of storage time changed the statistical results presented in this paper, we cannot rule out that storage time had a direct impact on the measured concentrations.

## Supporting information

S1 File (DOCX)

## References

[1] Dobson R, Giovannoni G. Multiple sclerosis–a review. Euro J of Neurology. 2019 Jan;26(1):27–40. doi: 10.1111/ene.13819 30300457

[2] Rocca MA, Valsasina P, Martinelli V, Misci P, Falini A, Comi G, et al. Large-scale neuronal network dysfunction in relapsing-remitting multiple sclerosis. Neurology. 2012 Oct 2;79(14):1449–57. doi: 10.1212/WNL.0b013e31826d5f10 22955126

[3] Høgestøl EA, Nygaard GO, Alnæs D, Beyer MK, Westlye LT, Harbo HF. Symptoms of fatigue and depression is reflected in altered default mode network connectivity in multiple sclerosis. PLoS One. 2019;14(4):e0210375. doi: 10.1371/journal.pone.0210375 30933977 PMC6443168

[4] Orsi G, Cseh T, Hayden Z, Perlaki G, Nagy SA, Giyab O, et al. Microstructural and functional brain abnormalities in multiple sclerosis predicted by osteopontin and neurofilament light. Multiple Sclerosis and Related Disorders. 2021 Jun;51:102923. doi: 10.1016/j.msard.2021.102923 33813096

[5] Rocca MA, Valsasina P, Absinta M, Riccitelli G, Rodegher ME, Misci P, et al. Default-mode network dysfunction and cognitive impairment in progressive MS. Neurology. 2010 Apr 20;74(16):1252–9. doi: 10.1212/WNL.0b013e3181d9ed91 20404306

[6] Huiskamp M, Eijlers AJC, Broeders TAA, Pasteuning J, Dekker I, Uitdehaag BMJ, et al. Longitudinal Network Changes and Conversion to Cognitive Impairment in Multiple Sclerosis. Neurology. 2021 Aug 24;97(8):e794–802. doi: 10.1212/WNL.0000000000012341 34099528 PMC8397585

[7] Goulden N, Khusnulina A, Davis NJ, Bracewell RM, Bokde AL, McNulty JP, et al. The salience network is responsible for switching between the default mode network and the central executive network: Replication from DCM. NeuroImage. 2014 Oct;99:180–90. doi: 10.1016/j.neuroimage.2014.05.052 24862074

[8] Wu L, Zhang Y, Zhou F, Gao L, He L, Zeng X, et al. Altered intra- and interregional synchronization in relapsing–remitting multiple sclerosis: a resting-state fMRI study. Neuropsychiatr Dis Treat. 2016 Apr 15;12:853–62. doi: 10.2147/NDT.S98962 27143886 PMC4841392

[9] Comi C, Cappellano G, Chiocchetti A, Orilieri E, Buttini S, Ghezzi L, et al. The Impact of Osteopontin Gene Variations on Multiple Sclerosis Development and Progression. Clinical and Developmental Immunology. 2012;2012:1–6. doi: 10.1155/2012/212893 23008732 PMC3447190

[10] Comabella M, Pericot I, Goertsches R, Nos C, Castillo M, Blas Navarro J, et al. Plasma osteopontin levels in multiple sclerosis. Journal of Neuroimmunology. 2005 Jan;158(1–2):231–9. doi: 10.1016/j.jneuroim.2004.09.004 15589058

[11] Orsi G, Hayden Z, Cseh T, Berki T, Illes Z. Osteopontin levels are associated with late-time lower regional brain volumes in multiple sclerosis. Sci Rep. 2021 Dec 8;11(1):23604. doi: 10.1038/s41598-021-03173-3 34880402 PMC8654976

[12] Thompson AJ, Banwell BL, Barkhof F, Carroll WM, Coetzee T, Comi G, et al. Diagnosis of multiple sclerosis: 2017 revisions of the McDonald criteria. The Lancet Neurology. 2018. doi: 10.1016/S1474-4422(17)30470-2 29275977

[13] Nieto-Castanon A, Whitfield-Gabrieli S. CONN functional connectivity toolbox: RRID SCR_009550, release 21 [Internet]. 21st ed. Hilbert Press; 2021 [cited 2023 Nov 24]. https://www.hilbertpress.org/link-nieto-castanon2021

[14] Friston K, editor. Statistical parametric mapping: the analysis of functional brain images. Repr. Amsterdam Heidelberg: Academic Press; 2008. 647 p.

[15] Urchs S, Armoza J, Moreau C, Benhajali Y, St-Aubin J, Orban P, et al. MIST: A multi-resolution parcellation of functional brain networks. MNI Open Res. 2019 Mar 5;1:3.

[16] Agah E, Zardoui A, Saghazadeh A, Ahmadi M, Tafakhori A, Rezaei N. Osteopontin (OPN) as a CSF and blood biomarker for multiple sclerosis: A systematic review and meta-analysis. Wiendl H, editor. PLoS ONE. 2018 Jan 18;13(1):e0190252. doi: 10.1371/journal.pone.0190252 29346446 PMC5773083

[17] Husain-Krautter S, Kramer JM, Li W, Guo B, Rothstein TL. The osteopontin transgenic mouse is a new model for Sjögren’s syndrome. Clinical Immunology. 2015 Mar;157(1):30–42. 25572532 10.1016/j.clim.2014.12.010 PMC4357545

[18] Mishima R, Takeshima F, Sawai T, Ohba K, Ohnita K, Isomoto H, et al. High Plasma Osteopontin Levels in Patients With Inflammatory Bowel Disease. Journal of Clinical Gastroenterology. 2007 Feb;41(2):167–72. doi: 10.1097/MCG.0b013e31802d6268 17245215

[19] Kariya Y, Kariya Y, Saito T, Nishiyama S, Honda T, Tanaka K, et al. Increased cerebrospinal fluid osteopontin levels and its involvement in macrophage infiltration in neuromyelitis optica. BBA Clinical. 2015 Jun;3:126–34. doi: 10.1016/j.bbacli.2015.01.003 26673877 PMC4661545

[20] Shimizu Y, Ota K, Ikeguchi R, Kubo S, Kabasawa C, Uchiyama S. Plasma osteopontin levels are associated with disease activity in the patients with multiple sclerosis and neuromyelitis optica. Journal of Neuroimmunology. 2013 Oct;263(1–2):148–51. doi: 10.1016/j.jneuroim.2013.07.005 23910387

[21] O’Regan A, Berman JS. Osteopontin: a key cytokine in cell‐mediated and granulomatous inflammation. Int J Experimental Path. 2000 Dec;81(6):373–90. doi: 10.1046/j.1365-2613.2000.00163.x 11298186 PMC2517746

[22] Murugaiyan G, Mittal A, Weiner HL. Increased Osteopontin Expression in Dendritic Cells Amplifies IL-17 Production by CD4+ T Cells in Experimental Autoimmune Encephalomyelitis and in Multiple Sclerosis. The Journal of Immunology. 2008 Dec 1;181(11):7480–8. doi: 10.4049/jimmunol.181.11.7480 19017937 PMC2653058

[23] Rittling SR, Singh R. Osteopontin in immune-mediated diseases. Journal of Dental Research. 2015 Dec 4;94(12):1638–45. doi: 10.1177/0022034515605270 26341976 PMC4681477

[24] Niino M, Kikuchi S. Osteopontin and multiple sclerosis: An update: OPN and MS. Clinical and Experimental Neuroimmunology. 2011 May;2(2):33–40.

[25] Rabenstein M, Vay SU, Flitsch LJ, Fink GR, Schroeter M, Rueger MA. Osteopontin directly modulates cytokine expression of primary microglia and increases their survival. Journal of Neuroimmunology. 2016 Oct;299:130–8. doi: 10.1016/j.jneuroim.2016.09.009 27725111

[26] Mohamed DL, Amer HA, Aboshady RA, Abdel Hafeez MA, Lotfy NM. Serum osteopontin as a blood biomarker in relapsing–remitting multiple sclerosis Egyptian patients. The Egyptian Journal of Laboratory Medicine. 2021;33(1):6–11.

[27] Sridharan D, Levitin DJ, Menon V. A critical role for the right fronto-insular cortex in switching between central-executive and default-mode networks. Proc Natl Acad Sci USA. 2008 Aug 26;105(34):12569–74. doi: 10.1073/pnas.0800005105 18723676 PMC2527952

[28] Meijer KA, Eijlers AJC, Douw L, Uitdehaag BMJ, Barkhof F, Geurts JJG, et al. Increased connectivity of hub networks and cognitive impairment in multiple sclerosis. Neurology. 2017 May 30;88(22):2107–14. doi: 10.1212/WNL.0000000000003982 28468841

[29] Battaglini M, Giorgio A, Stromillo ML, Bartolozzi ML, Guidi L, Federico A, et al. Voxel-wise assessment of progression of regional brain atrophy in relapsing-remitting multiple sclerosis. J Neurol Sci. 2009 Jul 15;282(1–2):55–60. doi: 10.1016/j.jns.2009.02.322 19286193

[30] Nocentini U, Bozzali M, Spanò B, Cercignani M, Serra L, Basile B, et al. Exploration of the relationships between regional grey matter atrophy and cognition in multiple sclerosis. Brain Imaging Behav. 2014 Sep;8(3):378–86. doi: 10.1007/s11682-012-9170-7 22584774

[31] Bizzo BC, Arruda‐Sanchez T, Tobyne SM, Bireley JD, Lev MH, Gasparetto EL, et al. Anterior Insular Resting‐State Functional Connectivity is Related to Cognitive Reserve in Multiple Sclerosis. Journal of Neuroimaging. 2021 Jan;31(1):98–102. doi: 10.1111/jon.12779 32857919

[32] Haider L, Zrzavy T, Hametner S, Höftberger R, Bagnato F, Grabner G, et al. The topograpy of demyelination and neurodegeneration in the multiple sclerosis brain. Brain. 2016 Mar;139(3):807–15. doi: 10.1093/brain/awv398 26912645 PMC4766379

