# Supplementary material for: Osteopontin predicts late-time salience network-related functional connectivity in multiple sclerosis
Source: PLoS One. 2024 Aug 29;19(8):e0309563. doi: 10.1371/journal.pone.0309563 (PMC11361605; doi:10.1371/journal.pone.0309563)
Supplement: S1 File — (DOCX) [file pone.0309563.s001.docx]

**Supplementary materials for:**

Osteopontin predicts late-time salience network-related functional connectivity in multiple sclerosis

Zsofia Kakucs^1,2^, Zsolt Illes^3,4,5^, Zsofia Hayden^3^, Timea Berki^6^, Gergely Orsi^3,7*^

^1^Department of Medical Imaging, Medical School, University of Pecs, Pecs, Hungary

^2^Department of Radiology and Medical Imaging, Mures County Emergency Clinical Hospital of Targu Mures, Targu Mures, Romania

^3^Department of Neurology, Medical School, University of Pecs, Pecs, Hungary

^4^Department of Neurology, Odense University Hospital, Odense, Denmark

^5^Department of Clinical Research, University of Southern Denmark, Odense, Denmark

^6^Department of Immunology and Biotechnology, Medical School, University of Pecs, Pecs, Hungary

^7^HUN-REN-PTE Clinical Neuroscience MR Research Group, Hungarian Research Network, Pecs, Hungary

^*^Corresponding author

E-mail: gergo.orsi@gmail.com

**1. Magnetic resonance imaging**

The following sequence parameters were used: MPRAGE (TR/TI/TE=2530/1100/3.37 ms; Flip Angle=7°; 176 sagittal slices; slice thickness=1 mm; FOV=256x256 mm^2^; matrix size=256x256; receiver bandwidth=200 Hz/pixel), FLAIR (TR/TI/TE=5000/1800/387 ms; Flip Angle=variable; 192 sagittal slices; slice thickness=0.9 mm; FOV=230x230 mm^2^; matrix size=256x256; receiver bandwidth=751 Hz/pixel), rs-fMRI (TR/TE=2000/30 ms; 36 axial slices; Flip Angle=76°; slice thickness=3 mm; number of volumes=300; FOV=210x210 mm^2^; matrix size=70x70; receiver bandwidth=2040 Hz/pixel).

**2. rs-fMRI evaluation**

Results included in this manuscript come from analyses performed using CONN1 (RRID:SCR_009550) release 21.a2 and SPM3 (RRID:SCR_007037) release 12.7771.

Preprocessing: Functional and anatomical data were preprocessed using a flexible preprocessing pipeline4 including creation of voxel-displacement maps, realignment with susceptibility distortion correction using fieldmaps, slice timing correction, outlier detection, indirect segmentation and MNI-space normalization, and smoothing. Functional data were realigned using SPM realign & unwarp procedure5 integrating fieldmaps for susceptibility distortion correction, where all scans were coregistered to a reference image (first scan of the first session) using a least squares approach and a 6 parameter (rigid-body) transformation and resampled using b-spline interpolation6 to simultaneously correct for motion, magnetic susceptibility geometric distortions, and their interaction. Temporal misalignment between different slices of the functional data (acquired in interleaved Siemens order) was corrected following SPM slice-timing correction (STC) procedure7,8, using sinc temporal interpolation to resample each slice BOLD timeseries to a common mid-acquisition time. Potential outlier scans were identified using ART9 as acquisitions with framewise displacement above 0.9 mm or global BOLD signal changes above 5 standard deviations10,11, and a reference BOLD image was computed for each subject by averaging all scans excluding outliers. Functional and anatomical data were normalized into standard MNI space, segmented into grey matter, white matter, and CSF tissue classes, and resampled to 2 mm isotropic voxels following an indirect normalization procedure11,12 using SPM unified segmentation and normalization algorithm13,14 with the default IXI-549 tissue probability map template. Last, functional data were smoothed using spatial convolution with a Gaussian kernel of 6 mm full width half maximum (FWHM).

Denoising: In addition, functional data were denoised using a standard denoising pipeline15 including the regression of potential confounding effects characterized by white matter timeseries (5 CompCor noise components), CSF timeseries (5 CompCor noise components), motion parameters and their first order derivatives (12 factors)16, outlier scans (below 26 factors)10, session effects and their first order derivatives (2 factors), and linear trends (2 factors) within each functional run, followed by bandpass frequency filtering of the BOLD timeseries17 between 0.008 Hz and 0.09 Hz. CompCor18,19 noise components within white matter and CSF were estimated by computing the average BOLD signal as well as the largest principal components orthogonal to the BOLD average, motion parameters, and outlier scans within each subject's eroded segmentation masks. From the number of noise terms included in this denoising strategy, the effective degrees of freedom of the BOLD signal after denoising were estimated to range from 81.3 to 89.9 (average 88.9) across all subjects11.

2.1 ROI-to-ROI analysis:

**First-level analysis:** ROI-to-ROI connectivity (RRC) matrices were estimated characterizing the functional connectivity between each pair of regions among 133 ROIs. Functional connectivity strength was represented by Fisher-transformed bivariate correlation coefficients from a general linear model (weighted-GLM20), estimated separately for each pair of ROIs, characterizing the association between their BOLD signal timeseries. In order to compensate for possible transient magnetization effects at the beginning of each run, individual scans were weighted by a step function convolved with an SPM canonical hemodynamic response function and rectified.

**Group-level analyses** were performed using a General Linear Model (GLM21). For each individual connection a separate GLM was estimated, with first-level connectivity measures at this connection as dependent variables (one independent sample per subject and one measurement per task or experimental condition, if applicable), and groups or other subject-level identifiers as independent variables. Connection-level hypotheses were evaluated using multivariate parametric statistics with random-effects across subjects and sample covariance estimation across multiple measurements. Inferences were performed at the level of individual clusters (groups of similar connections). Cluster-level inferences were based on parametric statistics within- and between- each pair of networks (Functional Network Connectivity22), with networks identified using a complete-linkage hierarchical clustering procedure23 based on ROI-to-ROI anatomical proximity and functional similarity metrics24. Results were thresholded using a combination of a p < 0.05 connection-level threshold and a familywise corrected p-FDR < 0.05 cluster-level threshold25.

2.2 Interhemispheric-correlation analysis:

**First-level analysis**: Interhemispheric correlation maps (IHC) characterizing the strength of homotopic functional connectivity between the two hemispheres were estimated as the Fisher-transformed bivariate correlation coefficient between the BOLD signal at each voxel and at the same anatomical location in the contralateral hemisphere (voxels with the same y&z MNI coordinates and opposite-sign x MNI coordinates)20,26.

**Group-level analyses** were performed using a General Linear Model (GLM21). For each individual voxel a separate GLM was estimated, with first-level connectivity measures at this voxel as dependent variables (one independent sample per subject and one measurement per task or experimental condition, if applicable), and groups or other subject-level identifiers as independent variables. Voxel-level hypotheses were evaluated using multivariate parametric statistics with random-effects across subjects and sample covariance estimation across multiple measurements. Inferences were performed at the level of individual clusters (groups of contiguous voxels). Cluster-level inferences were based on parametric statistics from Gaussian Random Field theory24,27. Results were thresholded using a combination of a cluster-forming p < 0.001 voxel-level threshold, and a familywise corrected p-FDR < 0.05 cluster-size threshold28.

2.3 Local correlation analysis:

**First-level analysis**: Local Correlation maps (LCOR) characterizing local coherence at each voxel were estimated as the weighted average of all short-range connections between a voxel and a 25 mm FWHM Gaussian neighborhood area29. Short-range connections were computed from the matrix of bivariate correlation coefficients between the BOLD timeseries from each pair of voxels, estimated using a singular value decomposition of the z-score normalized BOLD signal (subject-level SVD) with 64 components separately for each subject1.

**Group-level analyses** were performed using a General Linear Model (GLM21). For each individual voxel a separate GLM was estimated, with first-level connectivity measures at this voxel as dependent variables (one independent sample per subject and one measurement per task or experimental condition, if applicable), and groups or other subject-level identifiers as independent variables. Voxel-level hypotheses were evaluated using multivariate parametric statistics with random-effects across subjects and sample covariance estimation across multiple measurements. Inferences were performed at the level of individual clusters (groups of contiguous voxels). Cluster-level inferences were based on parametric statistics from Gaussian Random Field theory24,27. Results were thresholded using a combination of a cluster-forming p < 0.001 voxel-level threshold, and a familywise corrected p-FDR < 0.05 cluster-size threshold28.

2.4 ROI-TO-VOXEL analysis:

**First-level analysis:** Seed-based connectivity maps (SBC) were estimated characterizing the spatial pattern of functional connectivity with a seed area. Seed regions included 133 ROIs. Functional connectivity strength was represented by Fisher-transformed bivariate correlation coefficients from a weighted general linear model (weighted-GLM20), estimated separately for each seed area and target voxel, modeling the association between their BOLD signal timeseries. In order to compensate for possible transient magnetization effects at the beginning of each run, individual scans were weighted by a step function convolved with an SPM canonical hemodynamic response function and rectified.

**Group-level analyses** were performed using a General Linear Model (GLM21). For each individual voxel a separate GLM was estimated, with first-level connectivity measures at this voxel as dependent variables (one independent sample per subject and one measurement per task or experimental condition, if applicable), and groups or other subject-level identifiers as independent variables. Voxel-level hypotheses were evaluated using multivariate parametric statistics with random-effects across subjects and sample covariance estimation across multiple measurements. Inferences were performed at the level of individual clusters (groups of contiguous voxels). Cluster-level inferences were based on parametric statistics from Gaussian Random Field theory24,27. Results were thresholded using a combination of a cluster-forming p < 0.001 voxel-level threshold, and a familywise corrected p-FDR < 0.05 cluster-size threshold28.

## References

1 Whitfield-Gabrieli, S., & Nieto-Castanon, A. (2012). Conn: a functional connectivity toolbox for correlated and anticorrelated brain networks. Brain connectivity, 2(3), 125-141.

2 Nieto-Castanon, A. & Whitfield-Gabrieli, S. (2021). CONN functional connectivity toolbox: RRID SCR_009550, release 21. doi:10.56441/hilbertpress.2161.7292.

3 Penny, W. D., Friston, K. J., Ashburner, J. T., Kiebel, S. J., & Nichols, T. E. (Eds.). (2011). Statistical parametric mapping: the analysis of functional brain images. Elsevier.

4 Nieto-Castanon, A. (2020). FMRI minimal preprocessing pipeline. In Handbook of functional connectivity Magnetic Resonance Imaging methods in CONN (pp. 3–16). Hilbert Press.

5 Andersson, J. L., Hutton, C., Ashburner, J., Turner, R., & Friston, K. J. (2001). Modeling geometric deformations in EPI time series. Neuroimage, 13(5), 903-919.

6 Friston, K. J., Ashburner, J., Frith, C. D., Poline, J. B., Heather, J. D., & Frackowiak, R. S. (1995). Spatial registration and normalization of images. Human brain mapping, 3(3), 165-189.

7 Henson, R. N. A., Buechel, C., Josephs, O., & Friston, K. J. (1999). The slice-timing problem in event-related fMRI. NeuroImage, 9, 125.

8 Sladky, R., Friston, K. J., Tröstl, J., Cunnington, R., Moser, E., & Windischberger, C. (2011). Slice-timing effects and their correction in functional MRI. Neuroimage, 58(2), 588-594.

9 Whitfield-Gabrieli, S., Nieto-Castanon, A., & Ghosh, S. (2011). Artifact detection tools (ART). Cambridge, MA. Release Version, 7(19), 11.

10 Power, J. D., Mitra, A., Laumann, T. O., Snyder, A. Z., Schlaggar, B. L., & Petersen, S. E. (2014). Methods to detect, characterize, and remove motion artifact in resting state fMRI. Neuroimage, 84, 320-341.

11 Nieto-Castanon, A. (submitted). Preparing fMRI Data for Statistical Analysis. In M. Filippi (Ed.). fMRI techniques and protocols. Springer. doi:10.48550/arXiv.2210.13564

12 Calhoun, V.D., Wager, T.D., Krishnan, A., Rosch, K.S., Seymour, K.E., Nebel, M.B., Mostofsky, S.H., Nyalakanai, P. and Kiehl, K. (2017). The impact of T1 versus EPI spatial normalization templates for fMRI data analyses (Vol. 38, No. 11, pp. 5331-5342).

13 Ashburner, J., & Friston, K. J. (2005). Unified segmentation. Neuroimage, 26(3), 839-851.

14 Ashburner, J. (2007). A fast diffeomorphic image registration algorithm. Neuroimage, 38(1), 95-113.

15 Nieto-Castanon, A. (2020). FMRI denoising pipeline. In Handbook of functional connectivity Magnetic Resonance Imaging methods in CONN (pp. 17–25). Hilbert Press.

16 Friston, K. J., Williams, S., Howard, R., Frackowiak, R. S., & Turner, R. (1996). Movement-related effects in fMRI time-series. Magnetic resonance in medicine, 35(3), 346-355.

17 Hallquist, M. N., Hwang, K., & Luna, B. (2013). The nuisance of nuisance regression: spectral misspecification in a common approach to resting-state fMRI preprocessing reintroduces noise and obscures functional connectivity. Neuroimage, 82, 208-225.

18 Behzadi, Y., Restom, K., Liau, J., & Liu, T. T. (2007). A component based noise correction method (CompCor) for BOLD and perfusion based fMRI. Neuroimage, 37(1), 90-101.

19 Chai, X. J., Nieto-Castanon, A., Ongur, D., & Whitfield-Gabrieli, S. (2012). Anticorrelations in resting state networks without global signal regression. Neuroimage, 59(2), 1420-1428.

20 Nieto-Castanon, A. (2020). Functional Connectivity measures. In Handbook of functional connectivity Magnetic Resonance Imaging methods in CONN (pp. 26–62). Hilbert Press.

21 Nieto-Castanon, A. (2020). General Linear Model. In Handbook of functional connectivity Magnetic Resonance Imaging methods in CONN (pp. 63–82). Hilbert Press.

22 Jafri, M. J., Pearlson, G. D., Stevens, M., & Calhoun, V. D. (2008). A method for functional network connectivity among spatially independent resting-state components in schizophrenia. Neuroimage, 39(4), 1666-1681.

23 Sørensen, T. (1948). A method of establishing groups of equal amplitude in plant sociology based on similarity of species and its application to analyses of the vegetation on Danish commons. Biologiske Skrifter / Kongelige Danske Videnskabernes Selskab 5: 1-34.

24 Nieto-Castanon, A. (2020). Cluster-level inferences. In Handbook of functional connectivity Magnetic Resonance Imaging methods in CONN (pp. 83–104). Hilbert Press.

25 Benjamini, Y., & Hochberg, Y. (1995). Controlling the false discovery rate: a practical and powerful approach to multiple testing. Journal of the Royal statistical society: series B (Methodological), 57(1), 289-300.

26 Jin, X., Liang, X., & Gong, G. (2020). Functional integration between the two brain hemispheres: evidence from the homotopic functional connectivity under resting state. Frontiers in Neuroscience, 14.

27 Worsley, K. J., Marrett, S., Neelin, P., Vandal, A. C., Friston, K. J., & Evans, A. C. (1996). A unified statistical approach for determining significant signals in images of cerebral activation. Human brain mapping, 4(1), 58-73.

28 Chumbley, J., Worsley, K., Flandin, G., & Friston, K. (2010). Topological FDR for neuroimaging. Neuroimage, 49(4), 3057-3064.

29 Deshpande, G., LaConte, S., Peltier, S., & Hu, X. (2009). Integrated local correlation: a new measure of local coherence in fMRI data. Human brain mapping, 30(1), 13-23.
